# Study of analytical method to seek for exact solutions of variant Boussinesq equations

**DOI:** 10.1186/2193-1801-3-324

**Published:** 2014-06-27

**Authors:** Kamruzzaman Khan, M Ali Akbar

**Affiliations:** Department of Mathematics, Pabna University of Science and Technology, Pabna, 6600 Bangladesh; Department of Applied Mathematics, University of Rajshahi, Rajshahi, 6205 Bangladesh

**Keywords:** Enhanced (*G′/G*)-expansion method, Traveling wave, NLEEs, Variant Boussinesq equations

## Abstract

**Abstract:**

In this paper, we have been acquired the soliton solutions of the Variant Boussinesq equations. Primarily, we have used the enhanced (*G′/G*)-expansion method to find exact solutions of Variant Boussinesq equations. Then, we attain some exact solutions including soliton solutions, hyperbolic and trigonometric function solutions of this equation.

**Mathematics subject classification:**

35 K99; 35P05; 35P99

## Background

The theory of nonlinear evolution equations (NLEEs) has become one of the most important fields of study in mathematical physics and engineering. This is essentially due to the frequent occurrence of NLEEs in many branches of physics, mathematics, and other branches of sciences. With much interest and great demand for applications of NLEEs in various areas of sciences, several analytical methods to find exact solutions of NLEEs have been developed by diverse group of mathematician and physicist. It is significant that many equations of physics, chemistry, and biology contain empirical parameters or empirical functions. Exact solutions allow researchers to design and run experiments, by creating appropriate natural conditions, to determine these parameters or functions. A variety of powerful methods, such as the sine–cosine method (Wazwaz 2004a; Bekir 2008), The first integral method (Bekir et al. 2014; Jafari et al. 2012), the homotopy perturbation method (Mohyud-Din and Noor 2009; Mohyud-Din et al. 2011), the (*G′/G*)-expansion method (Wang et al. 2008; Zayed and Gepreel 2009; Guo and Zhou 2010), the Exp-function method (Bekir and Boz 2008; Akbar and Ali 2011; Naher et al. 2010; Ebadi et al. 2013), the modified simple equation method (Jawad et al. 2010; Zayed 2011; Khan and Akbar 2013a, [b]), the exp (−*Φ*(*ξ*))-expansion method (Khan and Akbar 2013c), the Enhanced (*G′/G*)-expansion Method (Khan and Akbar 2013d, [e]; Islam et al. 2014), the tanh-function method (Wazwaz 2004b, 2005, 2007), and the modified tanh–coth function method (Lee and Sakthivel 2011) were used to find new exact traveling wave solutions of nonlinear evolution equations in mathematical physics.

Various ansätz have been proposed for seeking traveling wave solutions of nonlinear differential equations. Recently, Wang et al. (2008) have introduced a simple method which is called the (*G*′/*G*) -expansion method to look for traveling wave solutions of nonlinear evolution equations, where *G* = *G*(*ξ*) satisfies the second order linear ordinary differential equation *G*″ + *λG*′ + *μG* = 0, where *λ* and *μ* are arbitrary constants and  be the traveling wave solution of NLEEs. By means of this method they have solved the KdV equation, the mKdV equation, the variant Boussinesq equations and the Hirota–Satsuma equations. Guo and Zhou (2010) have introduced an another method so called extended (*G*′/*G*)-expansion method where *G* = *G*(*ξ*) satisfies the second order linear ordinary differential equation *G*′′ + *μG* = 0, where , , *ξ* = *x* − *Vt*, *V* is a constant and  be the traveling wave solution.

They proposed extended (*G*′/*G*) -expansion method to construct travelling wave solutions of Whitham–Broer–Kaup–Like equations and coupled Hirota–Satsuma KdV equations.

Among those approaches, an enhanced (*G*′/*G*) -expansion method is a powerful tool to reveal more general solitons and periodic wave solutions of NLEEs in mathematical physics and engineering. The focal ideas of the enhanced (*G*′/*G*) -expansion method are that the traveling wave solutions of NLEEs can be expressed as rational functions of (*G*′/*G*), where *G* = *G*(*ξ*) satisfies the second order linear ordinary differential equation *G*′′ + *μ G* = 0.

The objective of this article is to present an enhanced (*G*′/*G*) -expansion method to construct the exact solitary wave solutions for NLEEs in mathematical physics via the Variant Boussinesq equations.

The article is arranged as follows: Methodology, Application of the enhanced (*G*′/*G*)-expansion method, Graphical representation, Comparisons and conclusions.

### Methodology

In this section, we discuss an analytical method, so called enhanced (*G′/G*)-expansion method, for deriving traveling wave solutions to PDEs. We will first discuss the method applied to a problem defined in terms of a nonlinear partial differential equation having two independent variables, one space dimension *x*, and another the time dimension *t*. Subsequently, it will be shown that the arguments extend naturally to coupled equations and also to problems defined in terms of two or more spatial dimensions, plus time.

Suppose the evolutionary equation, say in two independent variables *x* and *t*, for which we wish to find traveling wave solutions is given by
1

where *u*(*ξ*) = *u*(*x*, *t*) is an unknown function, *ℜ* is a polynomial of *u*(*x*, *t*) and its partial derivatives in which the highest order derivatives and nonlinear terms are involved. In the following, we give the main steps of this method (Khan and Akbar 2013a, 2013b; Islam et al. 2014):

Step 1. Combining the independent variables *x* and *t* into one variable *ξ*, we suppose that
2

where *V* ∈ *ℜ* − {0} is the wave velocity.

The traveling wave transformation Eq. (2) permits us to reduce Eq. (1) to the following ODE:
3

where *℘* is a polynomial in *u*(*ξ*) and its derivatives, while ,  and so on.

Step 2. We suppose that Eq. (3) has the formal solution
4

where *G* = *G*(*ξ*) satisfy the equation
5

in which *a*_*i*_, *b*_*i*_ (−*n* ≤ *i* ≤ *n*; *n* ∈ N) and *λ* are constants to be determined later, and *σ* = ± 1, *μ* ≠ 0.

Step 3. The positive integer *n* can be determined by considering the homogeneous balance between the highest order derivatives and the nonlinear terms appearing in Eq. (1) or Eq. (3). Moreover precisely, we define the degree of *u*(*ξ*) as *D*(*u*(*ξ*)) = *n* which gives rise to the degree of other expression as follows:
6

Therefore we can find the value of *n* in Eq. (4), using Eq. (6).

Step 4. We substitute Eq. (4) into Eq. (3) using Eq. (5) and then collect all terms of same powers of (*G*′/*G*)^*j*^ and  together, then set each coefficient of them to zero to yield an over-determined system of algebraic equations, solve this system for *a*_*i*_, *b*_*i*_, *λ* and *V*.

Step 5. From the general solution of Eq. (5), we get

When *μ* < 0,
78

Again, when *μ* > 0,
910

where *A* is an arbitrary constant. Finally, substituting *a*_*i*_, *b*_*i*_ (−*n* ≤ *i* ≤ *n*; *n* ∈ N), *λ*, *V* and Eqs. (7)-(10) into Eq. (4) we obtain traveling wave solutions of Eq. (1).

### Application

In this section we will exert the enhanced (G′/G)-expansion method to derive the exact traveling wave solutions of the Variant Boussinesq equations in the form (Wang et al. 2008)
1112

where *u* = *u*(*x*, *t*) represents the velocity and *H* = *H*(*x*, *t*) represents the total depth of water waves.

The traveling wave transformation equation *u* = *u*(*x*, *t*), *H*(*x*, *t*), *ξ* = *x* − *V t* converted Eq. (11) and Eq. (12) to the following ordinary differential equations for *u* = *u*(*ξ*) and *H* = *H*(*ξ*).
1314

Integrating Eqs. (13) and (14) once with respect to *ξ*, we obtain
1516

where *R* and *S* are constants of integration.

Now Eq. (16) yields,
17

Substituting Eq. (17) into Eq. (15), we get
18

The homogeneous balance between *u*″ and *u*^3^ yields *n* = 1.

Hence for *n* = 1, Eq. (4) reduces to
19

where *G* = *G*(*ξ*) satisfies Eq. (5). Substituting Eq. (4) along with Eq. (5) into Eq. (3), we get a polynomial of (*G*′/*G*)^*j*^ and . From these polynomials, we equate the coefficients of (*G*′/*G*)^*j*^ and , and setting them to zero, we get an over-determined system that consists of twenty-five algebraic equations. Solving this system for *a*_*i*_, *b*_*i*_, *λ* and *V*, we obtain the following values with the aid of symbolic computer software Maple 13.

Case 1: 

Case 2: 

Case 3: 

Case 4: 

Case 5: 

Case 6: 

Case 7: 

Case 8: 

Case 9: 

Case 10: 

Case 11: 

Case 12: 

Case 13: 

Case 14: 

Now putting the values of *a*_*i*_, *b*_*i*_, *λ* and *V* into Eq. (19) and Eq. (17), we obtain the following exact solutions.

Hyperbolic Function Solutions for *μ* < 0:

Family 1: 

where *ξ* = *x* − *a*_0_*t*.

Family 2: 

where *ξ* = *x* − *a*_0_*t*.

Family 3: 

where *ξ* = *x* − *a*_0_*t*.

Family 4: 

where *ξ* = *x* − *a*_0_*t*.

Family 5: 

where *ξ* = *x* − (2*μ λ* + *a*_0_) *t*.

Family 6: 

where *ξ* = *x* − (2*λμ* + *a*_0_) *t*.

Family 7: 

where *ξ* = *x* − (*a*_0_ − 2*λμ*) *t*.

Family 8: 

where *ξ* = *x* − (*a*_0_ − 2*λμ*) *t*.

Family 9: 

where *ξ* = *x* − *a*_0_*t*.

Family 10: 

where *ξ* = *x* − (2*μ λ* + *a*_0_) *t*.

Family 11: 

where *ξ* = *x* − (*a*_0_ − 2*λμ*) *t*.

Family 12: 

where *ξ* = *x* − *a*_0_*t*.

Family 13: 

where *ξ* = *x* − (*a*_0_ − *μ λ*) *t*.

Family 14: 

where *ξ* = *x* − (*μ λ* + *a*_0_) *t*.

Trigonometric Function Solutions for *μ* > 0:

Family 15: 

where *ξ* = *x* − *a*_0_*t*.

Family 16: 

where *ξ* = *x* − *a*_0_*t*.

Family 17: 

where *ξ* = *x* − *a*_0_*t*.

Family 18: 

where *ξ* = *x* − *a*_0_*t*.

Family 19: 

where *ξ* = *x* − (2*μ λ* + *a*_0_) *t*.

Family 20: 

where *ξ* = *x* − (2*λμ* + *a*_0_)*t*.

Family 21: 

where *ξ* = *x* − (*a*_0_ − 2*λμ*) *t*.

Family 22: 

where *ξ* = *x* − (*a*_0_ − 2*λμ*) *t*.

Family 23: 

where *ξ* = *x* − *a*_0_*t*.

Family 24: 

where *ξ* = *x* − (2*μ λ* + *a*_0_) *t*.

Family 25: 

where *ξ* = *x* − (*a*_0_ − 2*λμ*) *t*.

Family 26: 

where *ξ* = *x* − *a*_0_*t*.

Family 27: 

where *ξ* = *x* − (*a*_0_ − *μ λ*) *t*.

Family 28: 

where *ξ* = *x* − (*μ λ* + *a*_0_) *t*.

### Remark

All the obtained solutions have been checked with Maple by putting them back into the original equations and found correct.

### Graphical representations

Some of our obtained solutions are graphically represented in Figures 1, 2, 3, 4, 5, 6, 7, and 8.

**Figure 1:**
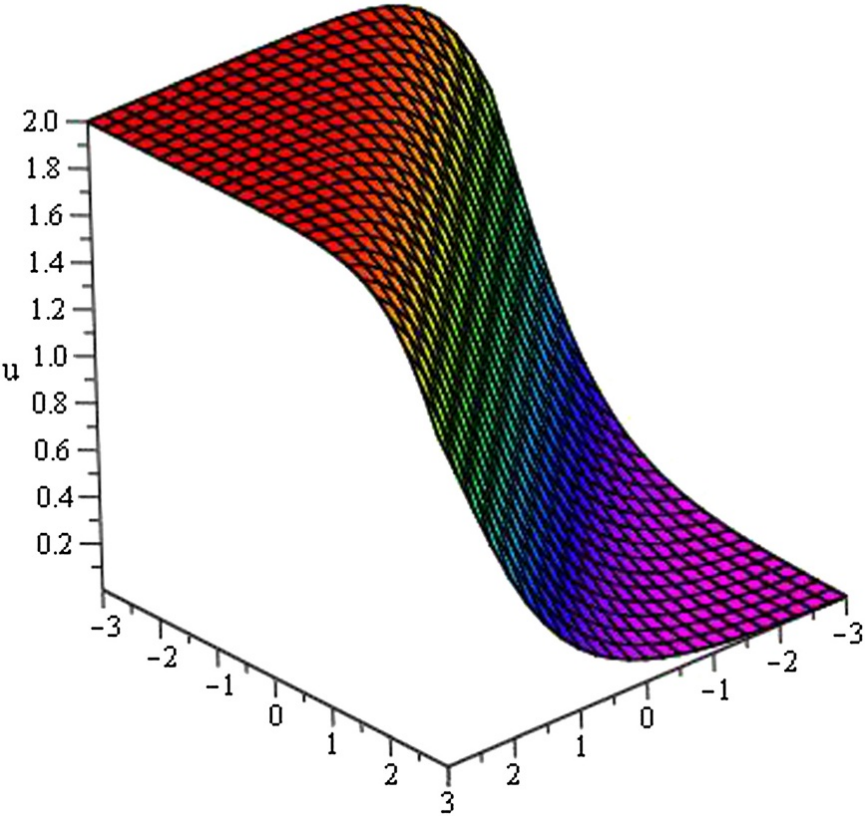
**Kink profile of** ***u***_**3**_ **(** ***ξ*** **)** **for** ***a***_**0**_ **= 1** **,** ***A =*** **0,** ***μ*** **= − 1** **within the interval** **− 3 ≤** ***x*** **,** ***t*** **≤ 3** **.**

**Figure 2:**
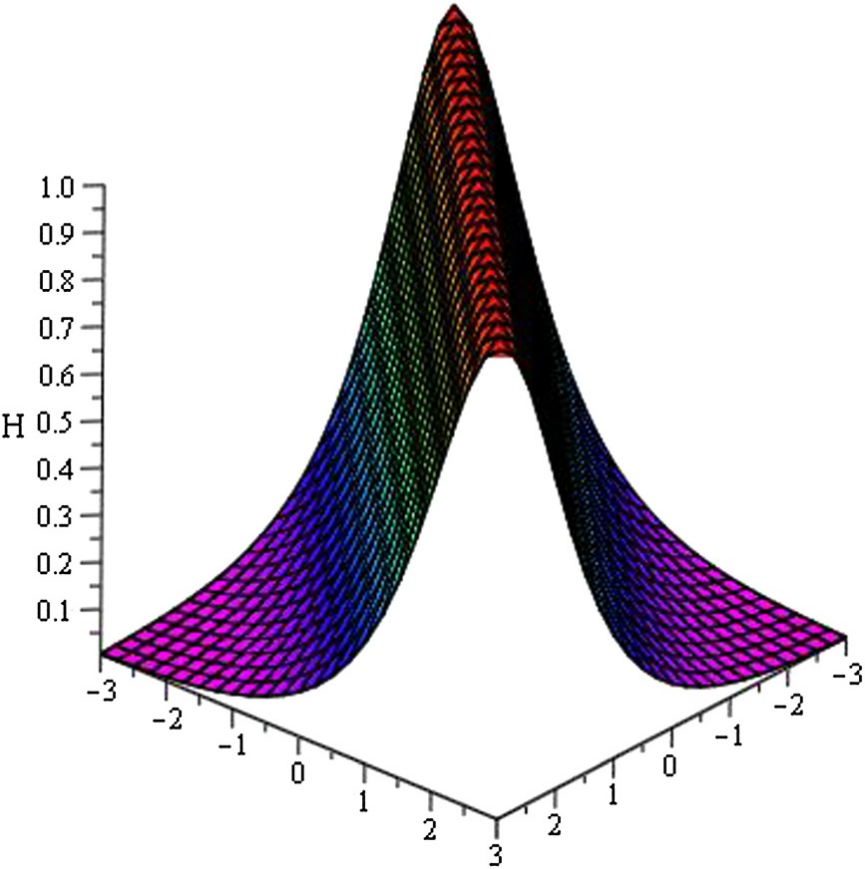
**Bell shaped soliton of** ***H***_**3**_ **(** ***ξ*** **)** **for** ***a***_**0**_ **= 1** **,** ***A =*** **0,** ***μ*** **= − 1** **within the interval** **− 3 ≤** ***x*** **,** ***t*** **≤ 3** **.**

**Figure 3:**
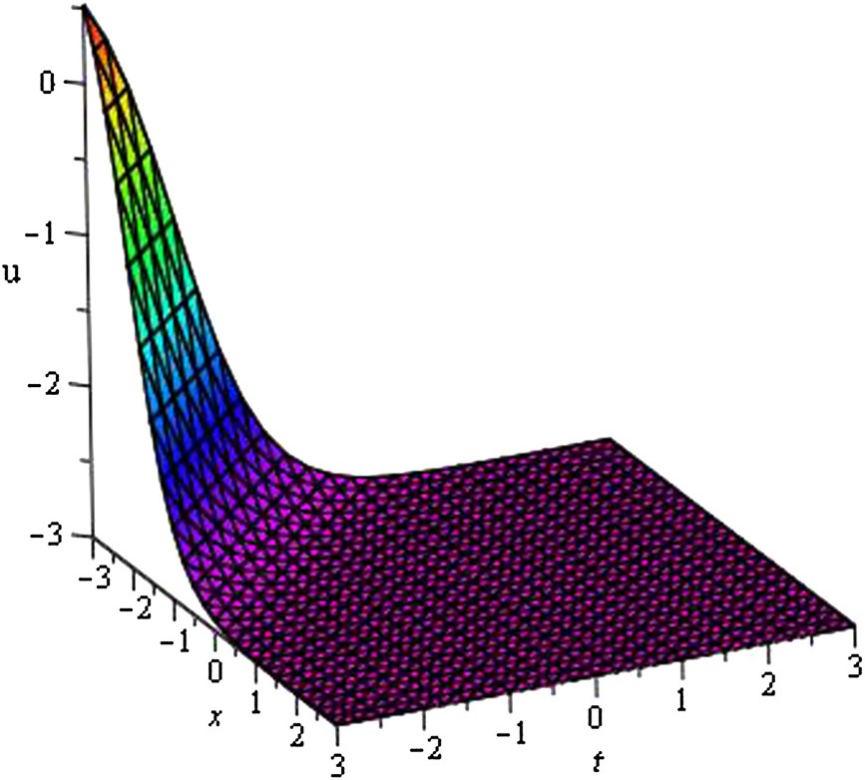
**Soliton profile of** ***u***_**8**_ **(** ***ξ*** **)** **for** ***a***_**0**_ **= 1** **,** ***A*** **= 5,** ***μ*** **= − 1** **,** ***λ*** **= 1** **within the interval** **− 3 ≤** ***x*** **,** ***t*** **≤ 3** **.**

**Figure 4:**
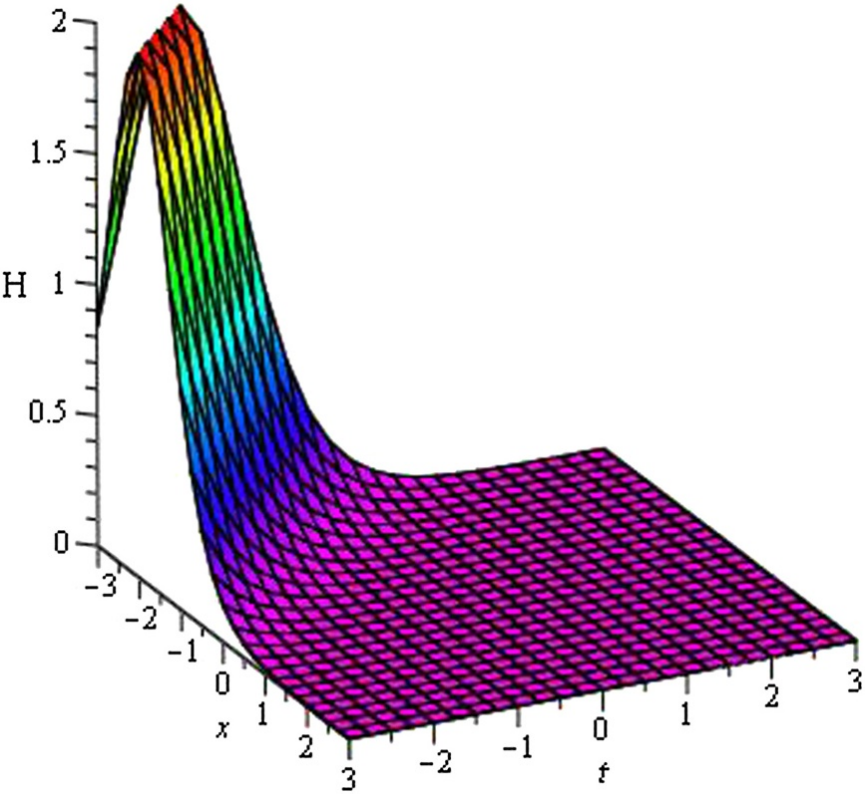
**Soliton profile of** ***H***_**8**_ **(** ***ξ*** **)** **for** ***a***_**0**_ **= 1** **,** ***A*** **= 5,** ***μ*** **= − 1** **,** ***λ*** **= 1** **within the interval** **− 3 ≤** ***x*** **,** ***t*** **≤ 3** **.**

**Figure 5:**
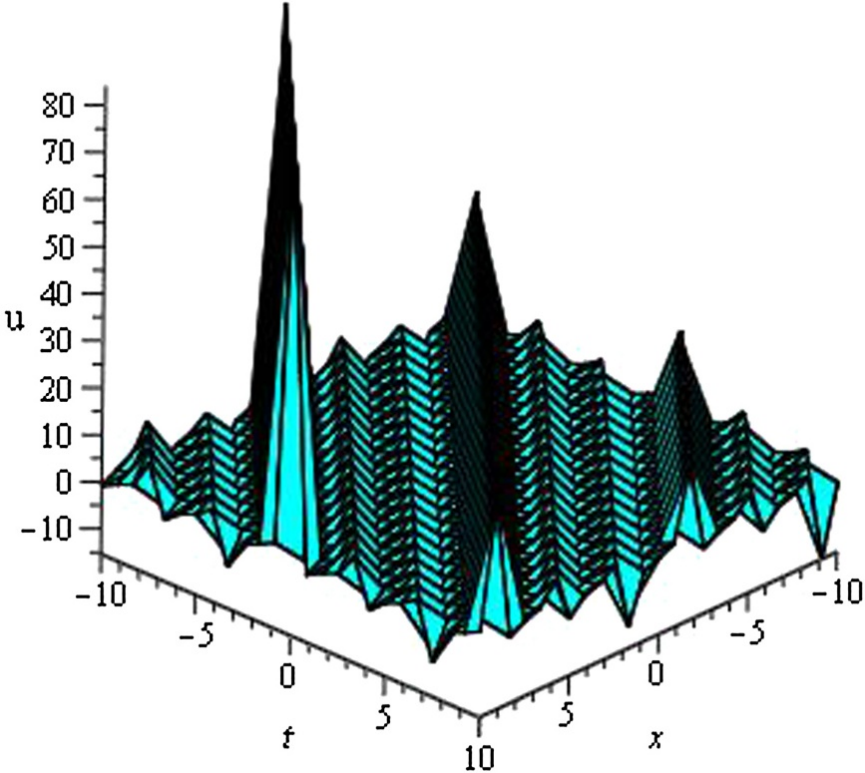
**Periodic wave of** ***u***_**31**_ **(** ***ξ*** **)** **for** ***a***_**0**_ **= 1** **,** ***A*** **= 0,** ***μ*** **= 3** **within the interval** **− 10 ≤** ***x*** **,** ***t*** **≤ 10** **.**

**Figure 6:**
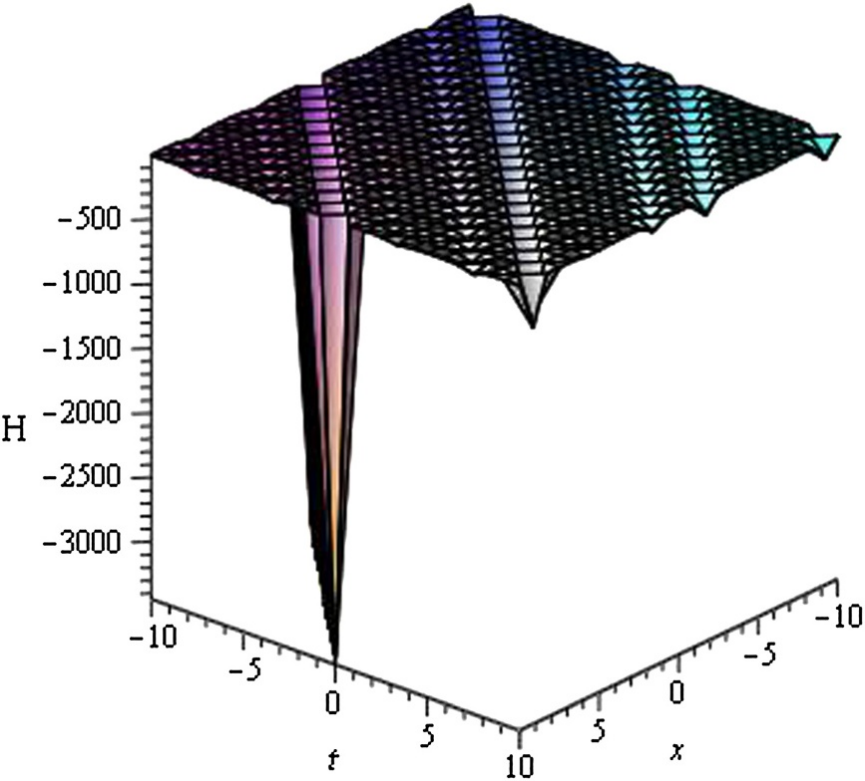
**Periodic wave of** ***H***_**31**_ **(** ***ξ*** **)** **for** ***a***_**0**_ **= 1** **,** ***A*** **= 0,** ***μ*** **= 3** **within the interval** **− 10 ≤** ***x*** **,** ***t*** **≤ 10** **.**

**Figure 7:**
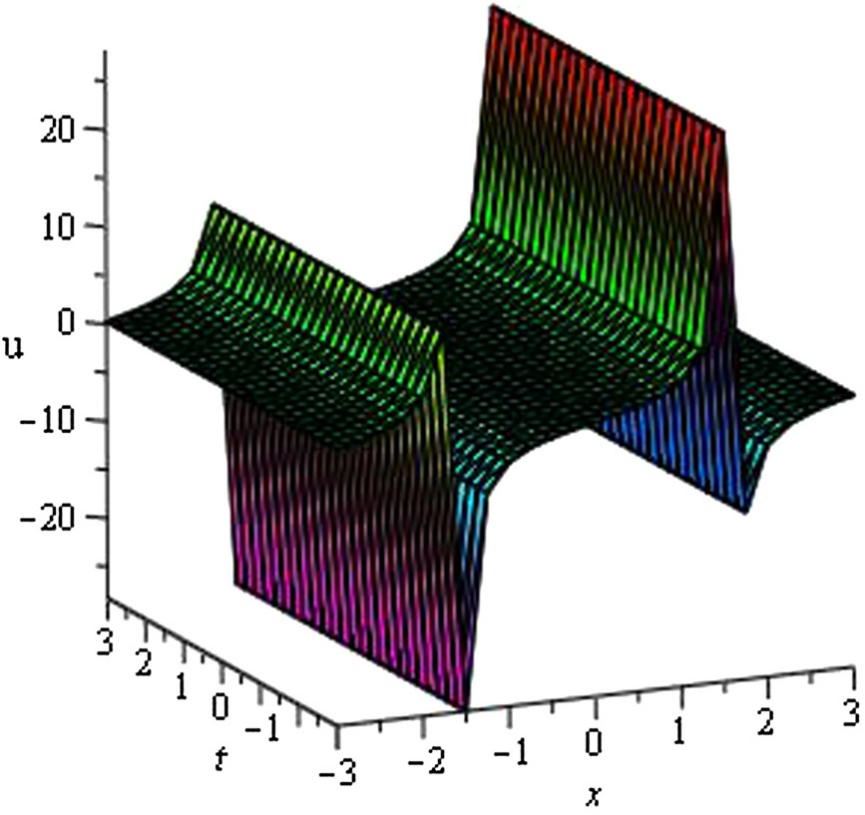
**Periodic wave of** ***u***_**38**_ **(** ***ξ*** **)** **for** ***a***_**0**_ **= 1** **,** ***A*** **= 0,** ***μ*** **= 1** **,** ***λ*** **= − 0.5** **within the interval** **− 3 ≤** ***x*** **,** ***t*** **≤ 3** **.**

**Figure 8:**
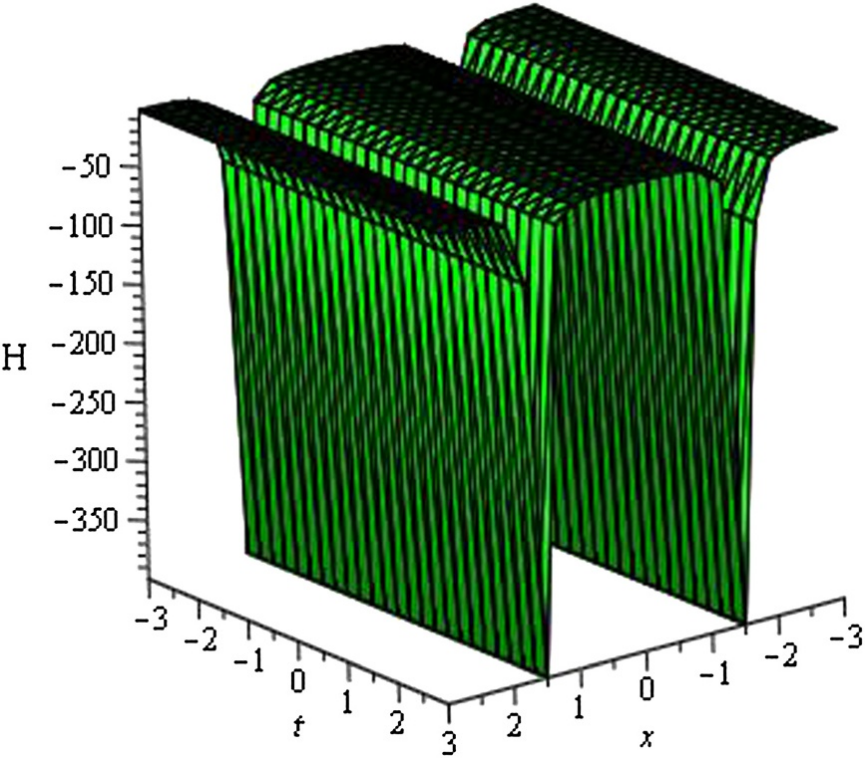
**Periodic wave of** ***H***_**38**_ **(** ***ξ*** **)** **for** ***a***_**0**_ **= 1** **,** ***A*** **= 0,** ***μ*** **= 1** **,** ***λ*** **= − 0.5** **within the interval** **− 3 ≤** ***x*** **,** ***t*** **≤ 3** **.**

### Comparisons with (*G*′/*G*) -expansion method

Wang et al. (2008) examined exact solutions of the Variant Boussinesq equations by using the (*G*′/*G*) -expansion method and obtained three solutions. On the contrary by using the enhanced (*G*′/*G*) -expansion method in this article we have obtained fifty six solutions. Furthermore, If we set *A* = 0, *a*_0_ = *β*_0_ then our solutions *u*_7_(*ξ*), *u*_11_(*ξ*) (Family 5) coincide with the solution Eq. (1.2) obtained by Wang et al. (2008) for *λ* = 0, *A*_1_ = 0 and if we set *A* = 0, *a*_0_ = *β*_0_ then our solutions *u*_8_(*ξ*), *u*_12_(*ξ*) coincide with the solution Eq. (1.2) obtained by Wang et al. (2008) for *λ* = 0, *A*_2_ = 0. Again if we set *A* = 0 then our solutions *H*_7_(*ξ*), *H*_11_(*ξ*) coincide with the solutions Eq. (1.1) obtained by Wang et al. (2008) for *λ* = 0, *A*_2_ = 0 and if we set *A* = 0 then our solutions *H*_8_(*ξ*), *H*_12_(*ξ*) coincide with the solutions Eq. (1.1) obtained by Wang et al. (2008) for *λ* = 0_,_*A*_2_ = 0. Correspondingly, for similar conditions our solutions of Family 19 and Family 21 are coincide with the solution Eq. (1.5) and Eq. (1.6) obtained by Wang et al. (2008). Rest of the solutions are freshly derived using enhanced (*G*′/*G*) -expansion method.

## Conclusions

Traveling wave solutions to nonlinear evolution equations arising in mathematical Physics are of theoretical importance. In this paper, the enhanced (*G′/G*)-expansion method has been successfully applied for obtaining exact traveling wave solutions of Variant Boussinesq equations. It has been shown that the enhanced (*G′/G*)-expansion method is quiet capable and well suited for finding exact solutions. The consistency of the method gives this method a wider applicability. With the aid of Maple and by putting them back into the original equation, we have assured the accuracy of the obtained solutions. Finally, it is worthwhile to mention that the method is straightforward and concise and it can be applied to other nonlinear evolution equations in engineering and the physical sciences.
